# Near complete genome sequences of African swine fever virus genotype II from domestic pigs in Indonesia, 2019–2020

**DOI:** 10.1128/mra.00236-25

**Published:** 2026-01-13

**Authors:** Sri Handayani Irianingsih, Lestari Lestari, Patrick Mileto, Yuli Miswati, Dilasdita Kartika Pradana, Suryo Purnomo Edi, Gemma Clark, Hendra Wibawa

**Affiliations:** 1 Disease Investigation Centre Wates, Directorate General of Livestock and Animal Health Services, Ministry of Agriculture, Kulon Progo, Indonesia; 2 CSIRO - Australian Centre for Disease Preparedness, Geelong, Australia; 3 Disease Investigation Centre Bukittinggi, Directorate General of Livestock and Animal Health Services, Ministry of Agriculture, Bukittinggi, Indonesia; 4 Disease Investigation Centre Denpasar, Directorate General of Livestock and Animal Health Services, Ministry of Agriculture, Denpasar, Indonesia; 5 Disease Investigation Centre Subang, Directorate General of Livestock and Animal Health Services, Ministry of Agriculture, Subang, Indonesia; 6 Directorate of Animal Health, Directorate General of Livestock and Animal Health Services, Ministry of Agriculture, Jakarta, Indonesia; Katholieke Universiteit Leuven, Leuven, Belgium

**Keywords:** ASF, genotype, whole genome sequence

## Abstract

The near complete genome sequences of African swine fever virus were reported from four samples originating from domestic pigs in Indonesia (Sumatera, Java, and Bali) from 2019 to 2020. Near complete genome sequence and phylogenetic analyses revealed that the genomes had an average length of 189,000 bp and belonged to genotype II.

## ANNOUNCEMENT

African swine fever (ASF) is a highly contagious viral disease in pigs caused by ASF virus (ASFV) (dsDNA virus; family *Asfarviridae*, genus *Asfivirus*) with mortality rates approaching 100% leading to huge economic impacts (1–3). ASF emerged in Asia when China reported in August 2018 (4). In 2019, it spread through mainland Asia, with the first case reported in Indonesia (5, 6) in North Sumatra in late 2019 (7). Subsequent cases were reported in Bali, Nusa Tenggara, and other islands (8), and it continues to circulate in Indonesia.

This study aimed to obtain complete genome profiles of representative samples in Indonesia and determine their relationships with other ASFVs in the region. From 2019 to 2020, symptomatic pig’s tissue and blood samples from three regions, Sumatera (*n* = 1), Java (*n* = 2), and Bali (*n* = 1), were tested to confirm ASFV via real-time PCR and those with CT value <25 were selected for whole genome sequencing.

Sequencing with MiSeq Reagent Kit v2 (300 cycles) on the Illumina MiSeq platform was conducted at the Disease Investigation Center Wates. DNA extraction used the QIAamp DNA extraction kit, concentrated and purified using the Zymo DNA Clean and Concentrator-5 kit. DNA concentrations were measured with the Qubit HS DNA assay. The Illumina Nextera XT DNA kit (Document #15031942 v07) was used per manufacturer’s instructions for sample library preparation incorporating 2 ng of DNA extract for tagmentation of genomic DNA, PCR amplification, and PCR clean-up. Geneious Prime 2022.2.2 was used for data analysis.

The data from the Illumina MiSeq were transferred to Geneious Prime for sequence trimming and mapping to a selected reference genome sequence ASFV/China/2018/AnhuiXCGQ (GenBank Accession No. MK128995) for genome assembly (9). In regions of low quality within the genome assembly, errors were removed, and a consensus sequence was generated. The final genome lengths were between 188,184 and 189,390 bp and the percentage of genome sequence was between 95.1% and 99.8% (Table 1).

**TABLE 1:** Details of the Indonesian ASFV genome sequences

Isolate	Genome size (bp)	Mean depth of coverage	No. of ORFs	% genome obtained	No. of Illumina reads	No. of mapped Illumina reads
ASFV/Indonesia/A02190909-4/2019	189,342	36×	177	99.6	12,125,198	26,725
ASFV/Indonesia/P04202068-51/2020	189,256	48×	179	99.6	6,796,356	37,344
ASFV/Indonesia/A06191713-8/2019	188,184	12×	158	95.1	10,703,816	9,321
ASFV/Indonesia/A08200012-09-11/2020	189,390	82×	177	99.8	11,902,114	60,508

Phylogenetic analysis of p72 and CD2v proteins, together with multiple sequence alignment of nucleotide (intergenic region [IGR]) and amino acid (central variable region [CVR]) markers showed that the Indonesian ASFV genomes sequenced in this study are classified as p72 genotype II (10), CD2v serogroup 8 (11), IGR variant II (12), and CVR subtype 1. These findings align with the characteristics of the pandemic strain circulating in Europe, China, and the Asia-Pacific region (13, 14) with the similarity of 95%–99% to each other.

## Data Availability

The genomes have been deposited in NCBI GenBank with accession numbers PP592870, PP592871, PP931992, and PP931993. The raw sequence data have been deposited in the NCBI Sequence Read Archive (SRA) database as SRX28409887, SRX28409884, SRX28409886, and SRX28409885, respectively, within the BioProject accession number PRJNA1251074.
